# From opiates to methamphetamine: building new harm reduction responses in Jakarta, Indonesia

**DOI:** 10.1186/s12954-019-0341-3

**Published:** 2019-12-11

**Authors:** Rafaela Rigoni, Sara Woods, Joost J. Breeksema

**Affiliations:** 1 Mainline Foundation, Amsterdam, Netherlands; 2 Department of History and Art History, Utrecht University, Utrecht, Netherlands; 3 University Center of Psychiatry, University Medical Center of Groningen, Groningen, Netherlands

**Keywords:** Methamphetamine, *Shabu*, Harm reduction, Stimulants, Outreach work, Indonesia, Community-based

## Abstract

**Background:**

Despite the rise of stimulant use, most harm reduction programs still focus on people who inject opioids, leaving many people who use methamphetamine (PWUM) underserviced. In Asia, especially, where methamphetamine prevalence has overtaken opioids prevalence, harm reduction programs assisting PWUM are rare. The few existing innovative practices focusing on methamphetamine use lie underreported. Understanding how these programs moved their focus from opiates to methamphetamine could help inspire new harm reduction responses. Hence, this paper analyzes a newly implemented outreach program assisting methamphetamine users in Jakarta, Indonesia. It addresses the program’s critical learning points when making the transition to respond to stimulant use.

**Methods:**

This case study is part of a more extensive research on good practices of harm reduction for stimulant use. For this case study, data was collected through Indonesian contextual documents and documents from the program, structured questionnaire, in-depth interviews with service staff and service users, a focus group discussion with service users, and in-loco observations of activities. For this paper, data was reinterpreted to focus on the key topics that needed to be addressed when the program transitioned from working with people who use opioids to PWUM.

**Results:**

Four key topics were found: (1) getting in touch with different types of PWUM and building trust relationships; (2) adapting safer smoking kits to local circumstances; (3) reframing partnerships while finding ways to address mental health issues; and (4) responding to local law enforcement practices.

**Conclusions:**

The meaningful involvement of PWUM was essential in the development and evaluation of outreach work, the planning, and the adaptation of safer smoking kits to local circumstances. Also, it helped to gain understanding of the broader needs of PWUM, including mental health care and their difficulties related to law enforcement activities. Operating under a broad harm reduction definition and addressing a broad spectrum of individual and social needs are preferable to focusing solely on specific interventions and supplies for safer drug use. Since many PWUM smoke rather than inject, securing funding for harm reduction focused on people who do not inject drugs and/or who do not use opioids is fundamental in keeping programs sustainable.

## Introduction

This paper presents a critical analysis of one of the seven case studies presented in a research about good harm reduction practices for people who use stimulants [1]. The present case is an outreach work project for PWUM in Jakarta, Indonesia, run by an NGO called Karisma. To our knowledge, this was the first harm reduction-oriented project in Southeast Asia focused on providing outreach work services for PWUM.

The present article explores and describes this case in more detail and pays special attention to the process of redirecting its harm reduction program from assisting people who inject opioids to assisting people who smoke methamphetamine. Nowadays, the project provides PWUM with oral information and leaflets on methamphetamine, mental health issues, drug use and use disorder, and health impacts of methamphetamine use. Karisma also distributes safer smoking kits and works on developing a network of services to address PWUM needs further.

From the critical analysis of this case study, four key topics arose that need to be addressed when a program transitions from working with people who use opioids to PWUM. These are [1] getting in touch with different types of PWUM and building trust relationships [2]; adapting safer smoking kits to local circumstances [3]; reframing partnerships while finding ways to address mental health issues; and [4] responding to local law enforcement practices.

The following pages of this section describe the context, including some background on the rise of stimulants use and the Indonesian case. A second section explains the methodology of the study. The two following sections describe and discuss the key learning points of Karisma’s PWUM harm reduction service when transitioning from opiates to methamphetamine. A final section concludes with the most relevant points harm reduction organizations need to pay attention to when operating similar transitions.

### The rise of stimulants and new harm reduction challenges

In recent years, several regions in the world have witnessed an increase in the use of stimulants. According to the World Drug Report 2018, amphetamine-type substances (ATS) are the second most commonly used illicit drug – after cannabis. ATS are a group of chemically and structurally related synthetic drugs that are powerful central nervous system stimulants. They increase the activity of the dopamine and noradrenaline neurotransmitter systems and raise levels of dopamine and norepinephrine in the brain [2]. It is estimated that around 34.2 million people have used ATS in the past year, ranging between 13 million and 58 million, and its use seems to be on the increase [3]. The International Drug Policy Consortium (IDPC) reports that civil society organizations, academics, NGOs, and international agencies all report increasing ATS use in every region of the world [4]. ATS are the dominant drug of choice in Asia [2], where methamphetamine prevalence has overtaken heroin prevalence since 2009 [5].

Methamphetamine is structurally similar to amphetamine, but it is more potent, and its effects typically last longer. On the illegal market, methamphetamine is sold in pill, powder, or crystalline forms. In East and Southeast Asia, methamphetamine in tablet form is common. These pills, generally called *yaba*, are typically of low purity and may contain several other (psychoactive) substances in addition to methamphetamine. While pills are generally taken orally, or sometimes crushed and smoked, the crystals – referred to as *shabu,* ice, or crystal meth – can be smoked or injected. Powdered methamphetamine is usually adulterated with an additional substance such as caffeine, dextrose, or lactose and can be taken orally, intranasally (snorted), or dissolved and injected.

Despite global increases in ATS use, evidence-based ATS-specific interventions remain underdeveloped [2]. In Southeast Asia, as in the rest of the world, most of the harm reduction services available in the region focus on people who inject opioids. Most traditional harm reduction interventions are funded under the umbrella of HIV prevention, focusing on interventions such as needle and syringe programs (NSP) and HIV testing and treatment. The development of such interventions has been particularly challenging in East and Southeast Asia. Although harm reduction has been accepted as a legitimate approach to addressing drug use in several Asian countries, the leading treatment for people who use ATS is compulsory abstinence-oriented treatment in residential centers. Human rights abuses have been reported in many of those centers, and the compulsory inpatient strategy lacks proof of effectiveness [6]. People who use ATS rarely use harm reduction services, largely because they do not identify with (problematic) opioid use. They often belong to different networks of users and thus do not perceive harm reduction services as relevant to them [6]. Besides, the use of stimulants brings new social and health challenges, and many existing harm reduction programs face the difficult dilemma of wanting to address an unassisted population but perceiving themselves as lacking the knowledge or resources to do so.

Fortunately, some innovative harm reduction practices to address stimulants exist brought about by harm reduction organizations rooted in the field. Atitude, for instance, is a housing first program for people using freebase cocaine in Brazil; El Achique is a drop-in center for people using cocaine base paste in Uruguay; COUNTERfit is a program distributing safer smoking kits for people using methamphetamine and/or freebase cocaine in Canada; Chemsafe offers an online intervention for people using stimulants while practicing chemsex in Spain; contemplation groups work to enhance self-regulation strategies of people using methamphetamine in South Africa; and various drug consumption rooms are open for people using freebase cocaine in the Netherlands. Programs like these have an in-depth knowledge of the context and the needs of the community of people who use drugs (PWUD). Nevertheless, many of these practices tend to remain unknown to the broader public, due to insufficient documentation and dissemination. Studies available (e.g. [7–9],) tend to focus on describing the achievements of such programs and pay less attention to the learning processes that organizations went through when developing such innovative practices. Understanding these learning processes can improve our knowledge of harm reduction efforts and may constitute a powerful tool to inspire other organizations to build new harm reduction responses.

### Indonesian context and Karisma’s shabu outreach case

Indonesia follows Asia’s regional trend of a rise in ATS use. According to UNODC estimates, methamphetamine is also the most widely used illicit drug in the country after cannabis, followed by heroin and MDMA [10]. Although its validity has been strongly criticized, the only national survey on drug use presents similar results to UNODC estimates, with methamphetamine, locally called *shabu*, as the second most popular drug in the country [11, 12]. Jakarta, Denpasar, Batam, Medan, and Makassar are the cities with the highest prevalence of methamphetamine use in Indonesia [9].

Indonesia’s drug policy is strictly prohibited and applies severe punitive measures to drug use, including the death penalty for drug trade, criminalization of substance use, and mandatory reporting on drug use [13]. The rights of PWUD are often violated by forced drug testing, detention, compulsory treatment, and extortion [14]. Despite the punitive regulations, harm reduction is legally supported [15], and harm reduction services are available throughout the country. The majority of these, however, continue to offer services for people who inject heroin only. NSP are offered by NGOs and primary health care services, and opioid treatment programs (OTPs) are carried out by public health services in primary health care clinics (called *Puskesmas*). The latest Global State of Harm Reduction report reveals 194 NSP sites and 92 OTP sites in the country [16]. In addition, 11 Indonesian prisons offer OTP, but no NSP is provided inside penitentiaries [16]. The case described in this study was the first harm reduction outreach work project in Southeast Asia to focus on stimulant drugs. The project is run by 11 paid staff members – 5 of which are outreach workers – and 17 voluntary outreach work peers in Jakarta. In 2018, the program had a yearly budget of €45.000, 90% of which provided by its international donor, a Dutch NGO called Mainline Foundation. The program operates since mid-2016 and is coordinated by Karitas Sani Madani Foundation (Karisma), a community-based organization set up in 2001 by people whose lives had been affected by problematic drug use. In 2004, the organization got international funding to provide outreach work for people who inject drugs (PWID) – who mainly used heroin – in Jakarta. In 2015, having run a solid NSP program for over a decade, the organization started noticing a drastic drop in the uptake of needles and syringes. If before they were distributing up to 20,000 needles a month, by 2015 the number was down to a couple of hundreds.

“We were asking ourselves what happened. It was so hard to find new people who used heroin. At the same time, we saw the rise of methamphetamine. And we really wanted to engage with and help people who use drugs.” (P6, male)

Up to 2015, national and international funding covered NSP programs only. When in 2015 international funding to work with PWUM was made available through Mainline, Karisma started developing the only project offering harm reduction for people who use *shabu* in Indonesia. A needs assessment [9] helped identify priorities for a pilot intervention: two drug hotspots in Jakarta, a focus on health consequences associated with methamphetamine use, and the specific harms caused by risky sexual behavior. Outreach work started in July 2016, and the team had to overcome the challenges of transitioning from reducing harms of opioids to reduce harms of methamphetamine use. The key learning points of this transition are the focus of this paper.

## Methodology

This article presents an in-depth exploration and critical analysis of a case study previously reported in a larger investigation [1]. This more extensive study, led by the first author, aimed at collecting and producing evidence of effective harm reduction interventions for people who use stimulants. In addition to a literature review on harm reduction interventions for people who use stimulants, seven case studies were described on good regional practices. Selection of these cases was based on a combination of the literature review and consultation with over 50 harm reduction projects and experts in more than 30 countries. The criteria of selection were available evidence on effectiveness; sustainability and/or cost-effectiveness of the project; projects’ potential for replicability; willingness to cooperate in the study; and being recognized as a good practice among harm reduction professionals and PWUD in its region. Karisma’s shabu outreach was the only project chosen in the Asian region.

The data collected for the original case study was used in the present article. Collection of data was carried out by the first author and followed the methodology described in the main study [1], consisting of the following components:

- Analysis of documents related to the project’s set up and development (project proposal, annual working plans, and narrative reports), unpublished studies referring to the project (needs assessment and a midterm assessment), and national and local policy documents and statistics related to drug use.
- An online structured questionnaire for management sent by email before the field visit, collecting data on the amount of PWUM assisted, finances, partners of the project, and services offered.
- Field observations focused on describing and understanding the local context, service providers’ activities, the relationship between service users and service providers, as well as any program specifics considered relevant by PWUM.
- In-depth interviews with eight service providers and two service users. These interviews addressed the objectives, activities, population assisted, network, successes, and challenges in assisting PWUM and future expectations of the program.
- A focus group discussion with ten service users, focusing on users’ perspectives on the program and the harms reduced regarding their use of stimulants since their participation in the program.

In-depth interviews and focus groups were audio-recorded and fully transcribed; field observations were typed-out. Qualitative data was analyzed using deductive thematic analysis [17]. Data from the structured questionnaire complemented the qualitative information concerning numbers of people assisted and funding. No review by a formal ethical committee was requested for the main study, as the type of involvement of participants did not fall under the Dutch act for medical and academic research with human subjects (WMO). To comply with ethical issues and data protection guidelines, participant organizations signed an informed consent form allowing for the disclosure of its data, to ensure a transparent and comprehensive description of their programs. Moreover, all interview and focus group discussion participants signed a consent form assuring their anonymity and had the right to withdraw from the study at any moment. All anonymized data was stored at a secured and backed-up server, only accessible to the research team. For the quotes used in the paper, each respondent is distinguished by a number, and SU refers to service user, while P refers to a professional working at the harm reduction program.

In the present article, data collected for Karisma’s project was critically re-read and reanalyzed to focus on another question, namely: *what are important learning points from the project regarding its transition from reducing harms for opioids injection to reducing harms for non-injecting stimulant use?* Although this was not the primary question in the main study, it emerged during the interviews, groups and observations done at Karisma as a critical feature in the project’s development. The second round of thematic analysis [17] was done focusing on these critical points in the program’s transitioning process, leading to the previously mentioned four key topics: (1) getting in touch with different types of PWUM and building trust relationships; (2) adapting safer smoking kits to local circumstances; (3) reframing partnerships while finding ways to address mental health issues; and (4) responding to local law enforcement practices. These topics are further described and discussed in the following sections.

## Results

### Key topic 1: getting in touch with different types of PWUM and building trust relationships

After the start of the outreach program in 2016, the first challenge the team faced was how to access PWUM. Both outreach workers and team coordinators were experienced in assisting people who inject opioids, but none had worked with PWUM before, nor did they have established links with this population’s networks. Not surprisingly, they felt they lacked sufficient knowledge about PWUM and their needs.

In the first 6 months (July to December 2016), the outreach team provided harm reduction services to a documented 194 individuals, ranging from 16 to 61 years old; 75% of these were male. Because initially, the team lacked staff with experience of *shabu* use, they had difficulties accessing PWUM. To address this, they added peer educators with lived experience of methamphetamine use to the team. The role of peer educators is to help outreach workers reach PWUM in their communities. The peer educators spread information and supply for safer drug use among people in their direct surroundings. The more experienced peer educators also helped the outreach team to open new spots for outreach work, based on their contacts in a specific area. Consequently, the outreach workers now cover hotspots in all districts of Jakarta.

At the time of this research, 17 people with lived experience of drug use were actively involved as peer educators or another voluntary project support. Peers explained that their role is to share their knowledge of *shabu* harm reduction with their friends and contacts and functioning as a role model.

“A lot of users are my friends and people I have interacted with in the past. I know that they are not living healthily... And that’s where I come in to give them some direction. […] I’ve experienced everything they have first-hand. They can relate to the experiences that I’ve had.” (P1, male)

Meaningfully involving peers in the program also helped the outreach team to better understand the needs and experiences of PWUM. At first, the outreach workers who were used to assisting people who use opioids found it very challenging to deal with this new population that focuses more on the benefits than on the risks of their drug use.

“Still for us working with heroin users is much easier[…] They have awareness, they realize: ‘I need help, I have a problem and I need help.’ In contrast, the shabu user doesn’t. [They think that] the heroin user is the one with problems. ‘I don’t get a withdrawal, I don’t experience withdrawal symptoms, I can still go to work, and I am still okay.’ Thus, our main challenge is to increase awareness about the health risks associated with shabu use.” (P6, male)

Peers helped the outreach team realize that it is not that people cannot see any harms caused by their use, or that they do not need any help, but that they have their reasons for focusing on the benefits of *shabu* use. Indeed, many PWUM interviewed for our case study said they like the effects of *shabu* as it enables them to be more active and productive.

“When you use shabu, you’re more focused, more diligent. Like when you have a lot of kids and you want to take care of everything yourself and you don’t have any help around the house. It makes you more productive.” (SU1, female)

Knowing this, Karisma’s team has been working with service users on the integration of the practical value of methamphetamine use into the development of their harm reduction interventions.

Thus, the meaningful involvement of PWUM in the program was a fundamental step towards understanding service users and building harm reduction strategies for PWUM. Karisma not only invited peers to join the outreach work but also to help plan, develop, and evaluate the program. The team developed mechanisms to increase their participation, such as inviting service users to the team’s weekly meetings. During these meetings, participants discuss the results of outreach strategies and try to find solutions for challenges. A lot of new ideas for the outreach approach come from the team’s weekly meetings. One of these ideas was the inclusion of a female outreach worker in the team. This female outreach worker provides separate assistance for women who do not feel comfortable mixing in groups with male service users. Karisma acknowledges women who use *shabu* that have separate needs.

“In my experience, women face higher risks in terms of meth use, as they are more vulnerable. They sell sex for money to buy meth more easily or become meth couriers and are taken advantage of; they are offered just a little bit of money or meth as a reward. When women are arrested, they are also more prone to exploitation by police. They are more closed and secretive in terms of their drug use. Sometimes they use it only around their close friends, even their husbands or their families don’t know about it.” (P4, female)

In 2017, the program expanded its interventions to cover hotspots of *shabu* use in all districts of Jakarta. In the same year, outreach reported reaching around 900 PWUM. Epidemiology and public health students from Atma Jaya University joined outreach workers during their activities and recorded the process. By comparing these fieldwork notes with the needs assessment [18], a local step-by-step guidebook on how to conduct outreach for people who use *shabu* was developed*.* Together with the university, outreach workers discovered that there are two generations of PWUM in the streets of Jakarta, each one with specific habits and networks. The “old generation” is formed by people around 35–40 years of age who are currently using *shabu*. They are former heroin (locally known as *putaw*) users who can no longer find heroin. The “younger generation” on the other hand, is formed by people around 14–28 years of age who never used heroin. Very often, *shabu* is the first illicit drug they have tried. Most PWUM from both the old and the young generation combine methamphetamine with one or more other substances to help them come down. Alcohol, cannabis, and benzodiazepines are the most common choices, with methadone also being used by former heroin users who are currently in OTP.

Knowledge of the differences between the two PWUM groups allowed for the program to provide for more appropriate harm reduction education. Many PWUM from the older generation are either currently in OTP or were previously assisted by a harm reduction service. Therefore, in comparison to the younger generation, they have more knowledge of care providers, blood-borne diseases, and safer sexual practices, as well as safer drug use practices. With them, outreach workers can focus on information and counseling related to the specifics of methamphetamine in comparison to heroin. They also address the heroin craving that many of these older generations PWUM have and educate them on harmful mixtures of uppers and downers. With the younger generation, outreach workers focus more on basic methamphetamine information and other drug effects, safer sexual practices, and safer drug use. They also include more information on where and how to search for (institutionalized) help, as the younger PWUM are not used to contacting care providers. Younger PWUM, according to the outreach team, are less inclined to sit and talk for a long time. Thus, they benefit from more creative and dynamic approaches. In one area, for instance, a peer educator is involving younger PWUM in doing volunteer jobs at their local community. It keeps them busy, and it helps to improve the relationship between PWUM and the community. Another outreach worker noticed that younger PWUM are often playing games on their mobile phones and started playing the same games to understand them better. He uses conversations related to the game’s strategies as a trigger for starting contacts or to improve general conversations and bonding.

Acknowledging the differences in the preferences of younger and older generations also allowed for more specific adaptations related to the distribution of supplies for safer drug use, as will be explained under topic 2.

### Topic 2: adapting safer smoking kits to local circumstances

**Fig. 1:**
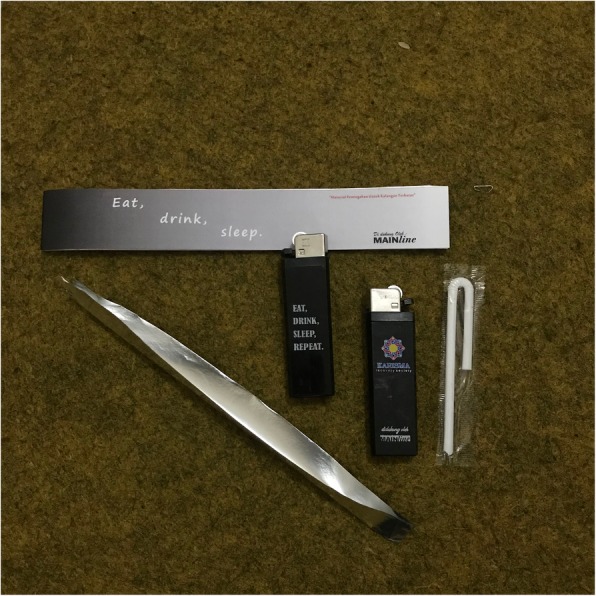
Safer smoking kits. Picture taken by the first author at Karisma’s office

Karisma started distributing safer smoking kits in 2017. These kits consist of a lighter, aluminum foil, straws, and informative leaflets (Fig. 1). Printed messages on the foil and the lighter – “eat, drink, and sleep” – function as a reminder of the importance of self-care*.* While distributing the kits, outreach workers and peer educators also provide harm reduction information.

**Fig. 2:**
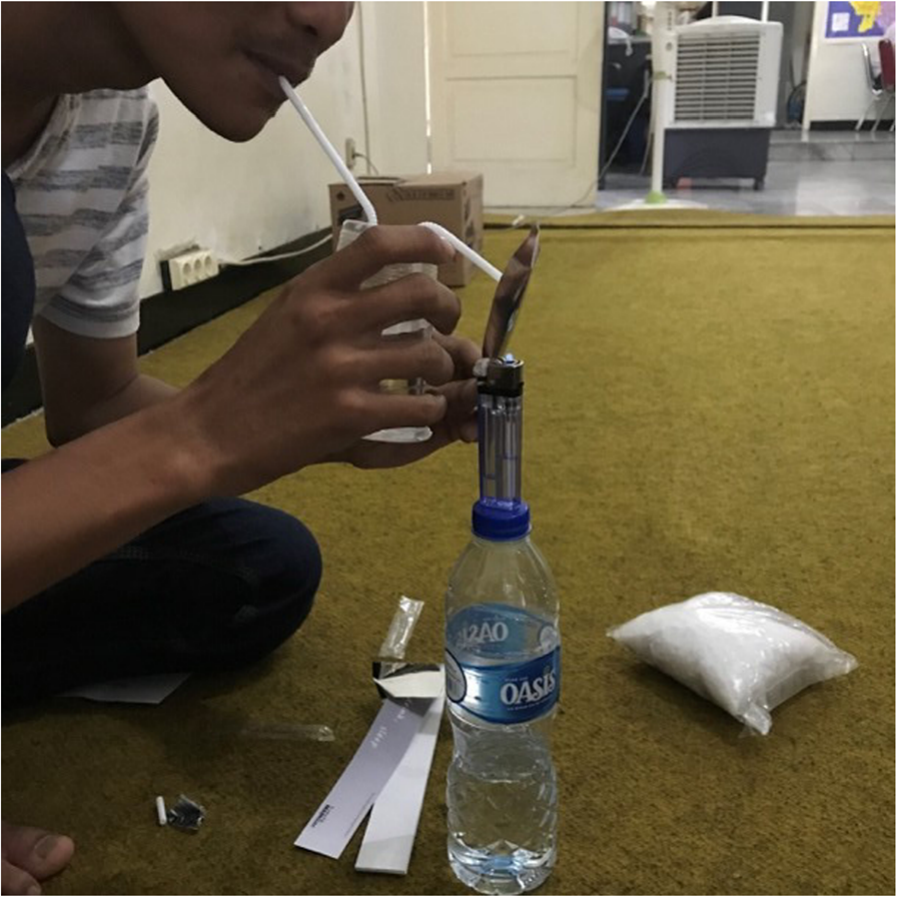
Home-made plastic bong and foil. Picture taken by the first author at Karisma’s office. It shows a peer outreach worker demonstrating how PWUM build and use their homemade bongs

**Fig. 3:**
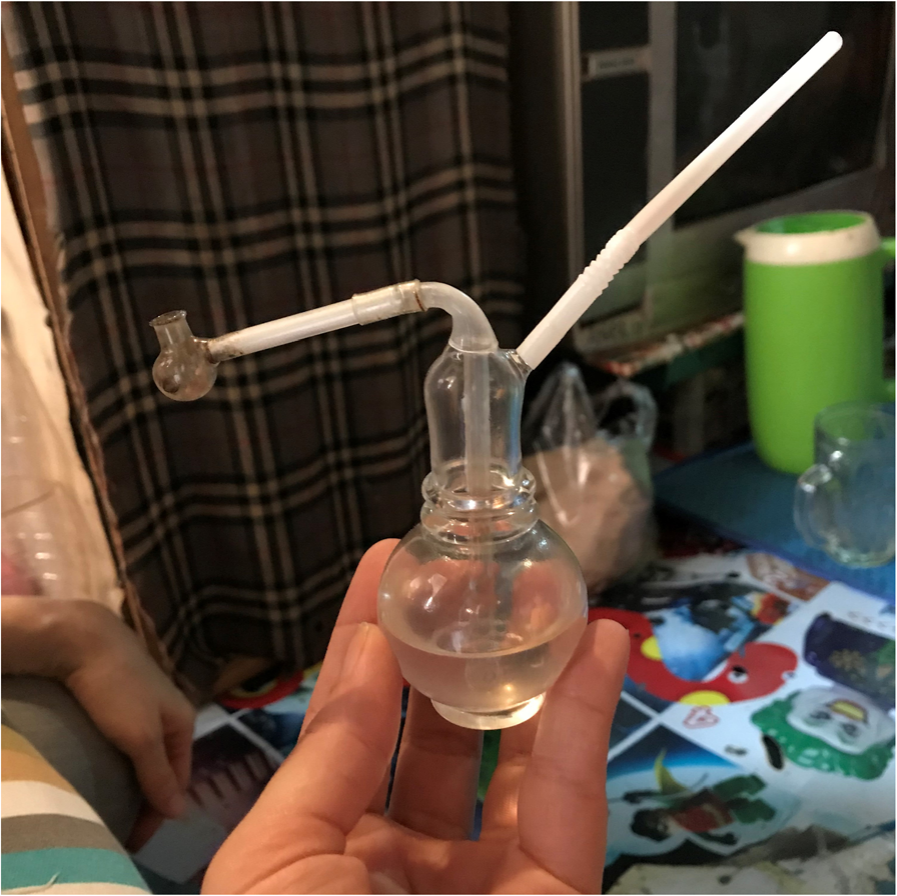
Glass bong with glass pipe. Picture taken by the first author during fieldwork observation, inside a room people rent to use *shabu*. Bongs like this can be rented at the place

The large majority of PWUM assisted by Karisma, from either generation, tend to smoke methamphetamine. The few cases of people injecting the drug are from people who were used to injecting their heroin. To smoke *shabu*, people normally use homemade bongs. Bongs are made from plastic cups or old bottles – such as small glass bottles of eucalyptus oil or plastic bottles – in which they make holes and attach a straw. PWUM prefer small bottles because it is easier to inhale the smoke. They generally prefer bongs over pipes as they feel the smoke is softer or less aggressive to inhale. The two generations tend to have different preferences when building their bongs. The young generation of users does not use foil (as in Fig. 2) but rather prefer a glass pipe (*cangklong*) (as in Fig. 3) or use a glass pipet (from ear medicine, for instance). Due to the strict drug regulations in Indonesia, carrying supplies for drug use can result in police harassment, along with the risk of being reported as a user and sent to forced drug treatment. In this context, oftentimes pipettes are preferred over glass pipes. They are less obviously linked to *shabu* use and, therefore, less risky when stopped by the police.

Both generations of PWUM tend to smoke *shabu* in groups, partially to reduce the cost of using. *Shabu* in Jakarta costs around IDR 200,000 (or €12^1^) a portion weighing 0.2 g. By pooling money together as a group, PWUM can assure a high for everyone. Sharing is part of a ritual not only related to drug use but also food and spaces. In this context, despite knowing the risks, PWUM admit to having difficulties not sharing their smoking equipment. For this reason, the staff would like to add a silicone mouthpiece to the distributed safer smoking kit. That way PWUM can still share bongs – they just have to replace the mouthpiece – and fewer bongs have to be carried around, potentially resulting in less police harassment. At the time of our study visit, however, Karisma had insufficient funding to buy such silicon mouthpieces. In consultation with service users, the team developed an alternative interim solution: the distribution of plastic straws in the kits. PWUM were already using plastic straws to build their homemade bongs, usually stolen from juice boxes sold at convenience stores. Commonly, people would share straws among the group, whether they are smoking in a bong or chasing the dragon (smoking on foil). With a wider distribution of straws – together with harm reduction information – the program aims to increase the individual use of straws and to decrease risks of sharing smoking equipment. Plastic straws are not the ideal instrument to smoke methamphetamine, as the plastic can burn, and people can inhale toxic vapors. However, budgets are limited, and some PWUM are so used to their smoking methods that switching to a potentially less harmful method can be complicated. Thus, teaching harm reduction methods that can be applied to their more harmful pipes is a pragmatic harm reduction alternative [19].

Addressing sharing cultures and unhealthy habits requires more than the distribution of safer smoking kits alone, and the outreach team tries to address this during their visits to the areas where PWUM get together to use the drug, so-called hotspots. The outreach workers have found out that giving a voice and space to those PWUM who refuse to share is an effective strategy contributing to gradual change. Service users reflect that, in their cultural context, not sharing involves developing sensitivity skills to be able to say no without offending others. By discussing their strategies on how they deal with these issues, they manage to inspire others not to share as well.

Thus, the content of the kits and accompanying harm reduction message was adapted to local circumstances and the users’ preferences and needs, to assure the effectiveness of this intervention. Important factors here were the differences between younger and older generation preferences as well as the legal context.

### Topic 3: reframing partnerships while finding ways to address mental health issues

Karisma’s staff reported that the available services for people who use drugs are generally not able to support PWUM. Thus, they aim to build a network with care providers, who could assist PWUM through various services, especially around mental health issues.

“Even though there are some services for people who use drugs, they don’t necessarily target shabu users. For example, we have around 18 to 20 primary health clinics that provide services for people using drugs. If we try to bring a shabu user, they go like: ‘okay, what do I do with this?’. (P6, male)

Some of the previously established partnerships for projects working with people who inject heroin also run effectively for PWUM. For instance, users can be referred to TB and HIV testing, counseling, and treatment. Those who also use heroin can get access to methadone or buprenorphine in OTP, and people who inject have access to NSP. People who would like to stop using can easily access drug treatment for rehabilitation, even though most drug treatment centers in Indonesia do not work with evidence-based treatment models. Karisma also runs its rehabilitation center and refers PWUM who are motivated to quit to this center. According to Karisma’s team, these are usually people who are on the brink of getting arrested or having severe problems with their family.

The most challenging partnerships are with law enforcement and mental health agencies. A significant and long-lasting challenge is the relationship with the police.

“The narcotics police are difficult to reach out to. We invite them to our events, but they never show up. […] They don’t want to be known. These narcotics police mostly work undercover.” (P3, male)

Repeated police harassment continues to happen and creates a lot of stress and distrust in the lives of PWUM. This is discussed in more detail under topic 4.

Assuring mental health care for PWUM is another main challenge for this new project. One of the problems is that, according to our interviewees, the average person in Indonesia does not understand what mental health services entail. Most people associate mental health with insanity and do not understand or talk about depression, anxiety, or stress in these terms.

“If we offer mental health information or services, they refuse and say: ‘I’m not crazy’.” (P4, female)

Most of Karisma’s service users do not understand the concept of mental health or recognize themselves as having mental health problems, even though virtually all of them mentioned becoming paranoid and being *very emotional* after having used *shabu*. To get more insight into how they may approach mental health issues, the outreach team started organizing focus group discussions with PWUM. They found that people were experiencing effects like paranoia and hallucinations but tended to ignore these issues.

An added challenge here is that mental health care services in Jakarta are not prepared to work with PWUM. The few times in which people searched for, help was not available.

“Every time they want to get counseling, or if they want to talk about their feelings or problems, it’s difficult for them to find people. If they approach health facilities, normally the health workers do not have enough information for them. […] most counseling services are not well-equipped for shabu users – only for heroin users. They don’t have the knowledge in terms of counseling for shabu.” (P3, male)

To address this challenge, in 2018, the outreach team planned nine informative events to discuss mental health at different health facilities. Local doctors were invited to talk to PWUM about mental health and the types of services offered by the centers. Outreach workers brief the doctors beforehand on the appropriate language to use with the population. At the time of the research, two of these meetings had happened. Between 15 and 20 PWUM were present in each session. This generated some positive results as it helped people recognize potential mental health issues:

“They attended meetings about mental health and realized that shabu users can also have mental health issues. They realized that what they experienced was in fact related to mental health. That was new for them.” (P4, female)

Nevertheless, the meetings were only partially successful. From the service users’ perspective, the presentation was not very attractive or comprehensive. From the staff’s perspective, doctors did inform participants on the services available but did not talk about the procedures and processes of accessing mental health services, such as costs and registration. Services can be free of charge if people have national health insurance, but most PWUM in Indonesia do not because they do not have the necessary legal documents (e.g., ID, family card, and residence register). According to Karisma staff, social workers are hard to find in Jakarta. There are few, and they mostly work from inside the ministries instead of close to the field. Many PWUM do not know where to search for social support, and outreach workers have limited time to accompany PWUM to health services.

At the time of this study, the Karisma team was in the process of organizing a partnership with the national Ministry of Health and the Provincial Health Department to discuss counseling issues for PWUM. The country has national guidelines for harm reduction, which do not include ATS, and Karisma expressed the need to include specific issues for PWUM in these guidelines. Karisma, the Ministry of Health, representatives of the Atma Jaya University, the 18 primary health care facilities which have Compulsory Reporting Institutions^2^, and counselors for addiction were all invited to an initial meeting to kickstart the partnership.

Additional steps have been taken by Mainline and Atma Jaya, which have worked in 2018 on a training for physicians and primary health care staff. Together with Karisma, these organizations are lobbying for integration of mental health care and support for people who use *shabu* in the primary health care system. In this process, the recognition of mental health symptoms and appropriate public health staff responses need to be negotiated. Besides, PWUM definitions of mental health need to be considered without blindly enforcing medical classifications.

### Topic 4: responding to local law enforcement practices

A significant challenge in Indonesia, affecting most of Karisma’s activities and the lives of PWUD, is the local law enforcement practices, especially, the conflict between criminalization of drug use on the one hand and state-level endorsement of harm reduction on the other.

A harsh police approach creates much mistrust among PWUM. The PWUM interviewed for this study all said to have been arrested at least once or to know someone who has been arrested because of drug use. Moreover, all are afraid of being reported by/to the police. Undercover narcotics police officers infiltrate user groups to find dealers and report users to rehab, in an effort to curb the trade and use of methamphetamine. The fear of being reported to the narcotics police provides outreach workers and peer educators with challenges; people are generally reluctant to allow newcomers into their drug-using circles.

“Right now, shabu use is booming, but this is also becoming more known among the police. The police want to prevent people from using shabu, so they might stop a stranger and ask: ‘do you use shabu?’ [as outreach work would do]. People will never say ‘yes.’ They will say: ‘what are you doing?! Are you a policeman?!’ There is always suspicion.” (P7, male)

Consequently, extra time needs to be invested in establishing a trust relationship with new contacts.

“It doesn’t happen instantly, getting someone to open up. Sometimes you just come and they (PWUM) immediately leave (out of suspicion). So, to really get that person involved and really want to listen to you, that takes time.” (P1, male)

In the current legal situation, human rights and legal protection for PWUM are of vital importance. Previous contact with human rights and legal organizations had already been established for people who inject opioids, and these also work for PWUM. Karisma collaborates with the Indonesian Drug Users Network (PKNI) and a Community Legal Aid Institute (LBHM). They refer PWUM who get caught with a small amount of *shabu* but are still prosecuted as dealers to these partners. Article 127 of the National Narcotics Law states that possession of less than a gram of *shabu* is considered to be for personal use. According to staff and service users, however, when somebody gets arrested with a small quantity of drugs (e.g., one package of 0.2 g), they are often charged with Article 114, which is intended for drug dealers.

“In Indonesia, drug-related cases are used by the police to get money. The police would file a charge against you, for instance with Article 114, but then they would make an offer: ‘do you want to be charged with Article 127 instead of 114? If so, you need to pay me with a price of a car’. Yeah… Indonesia. That’s very expensive.” (P5, male)

When PWUM get caught, outreach workers can also refer their family members to PKNI for more information about the situation. At PKNI or LBHM, they will be asked about the background of the person: whether they have undergone drug treatment for rehabilitation or whether they have accepted any health services. They collect proof that the person is indeed someone who uses drugs and not a dealer.

Strict law enforcement increases the mental health burden of PWUM. The threat of being caught promotes feelings of paranoia among service users. Moreover, *shabu* use tends to increase feelings of paranoia.

“You get paranoid that everyone can be somebody who reports you to the police.” (SU7, male)

“We’re tired of having all this paranoia. Sometimes we hallucinate and think ‘oh is there somebody there at the door?’ But there’s no one. So, if we see somebody we don’t know, we get paranoid” (SU4, female)

In order to avoid police attention, PWUM prefer to use *shabu* indoors, which has led to a market of room rentals. In some drug use hotspots, there are rooms where people can both buy and use *shabu*; other rooms are strategically located close by a dealer spot and are rented out just for *shabu* use. Outreach workers adapted their fieldwork to work more closely with the people renting out these spaces. The team reaches out to the landlord and gives him/her safer smoking kits and safer injection packs, as well as information and leaflets on harm reduction. These landlords get in touch with many PWUM daily and can become a contact point for spreading information on safer drug use.

## Discussion

In the process of adapting Karisma’s harm reduction program from a focus on opioids to an emphasis on methamphetamine, both old and new challenges arose. The lack of knowledge of the initial outreach work team regarding the experiences and needs of PWUM was the first challenge met by the team, which they managed to overcome by meaningfully involving peers. The practice of involving peers in outreach work is considered to be very effective to engage PWUS [20] and other marginalized and hard-to-reach populations [21, 22]. Peers are trusted more easily because they share norms, experiences, language, and background. This makes it easier to convey honest harm reduction education and information [23, 24]. Peer outreach is known to be particularly effective for safer drug use education and distribution of supplies [25]. There is a growing recognition of the need for more meaningful involvement of community members in public health programming [26]. Peer-led PWUD advocacy groups such as the International Network of People Who Use Drugs (INPUD) have been calling for more meaningful involvement of PWUD [27]. Particularly for services that need to consider new user groups such as people who smoke methamphetamine, including beneficiaries in a meaningful way is a crucial strategy. This means not only having people working in service delivery as (voluntary) peers but also involving them in the whole program set up and evaluation.

Strict local drug policies and law enforcement practices which harm(ed) people who use opioids continued to harm PWUM. The growing demand and availability of methamphetamine have also led to specific practices such as undercover police in user groups, which has increased distrust among PWUM and posed challenges for a starting outreach program. This threat has exacerbated paranoid episodes experienced by PWUM and in general increased the mental health burden already imposed by the use of methamphetamine.

Intensive police interventions and the specific preferences of the younger generation of PWUM have both challenged the outreach team to adapt the safer smoking kits they distribute. Adapting safer smoking kits to local circumstances and users’ preferences and needs increases acceptance of safer smoking equipment and prevents PWUM from continuing to use self-made pipes [28]. Sharing safer smoking paraphernalia, for instance, is not just an Indonesian phenomenon and has been well documented elsewhere. Methamphetamine use often takes place in a group setting where sharing is common, part of the culture, and not the result of an inability to buy or access new and clean supplies [29]. Even when using safer smoking kits, people may continue to share pipes for several personal and social reasons [28, 30]. These reasons include unfamiliarity with services; experiencing craving and feeling the compulsion to use immediately; being gifted drugs or pipes; or occasional smokers who do not carry the right equipment [31–35]. Especially for females, the sharing of pipes is also frequently the result of power relations, which renders them vulnerable [31, 36].

The context of strict policing in Indonesia often discourages PWUM from adopting safer smoking practices such as carrying glass bongs or glass pipes. When PWUM avoid carrying pipes for fear of police intervention, distributing mouthpieces can be a good and affordable harm reduction alternative. When PWUD resist adopting safer smoking kits, teaching harm reduction methods that can be applied to their more harmful pipes is a pragmatic harm reduction alternative [19]. Thus, even though not ideal, Karisma’s outreach team solution of distributing plastic straws is a pragmatic temporary solution, a compromise between respecting users’ habits and preferences, reducing harms and coping with lack of funding.

To protect PWUM against street harassment and the dangers of being reported by/to the police, Karisma’s outreach workers stimulate users’ strategy to use in rooms rented out, especially for drug use. This is a pragmatic harm reduction solution in the current political and cultural circumstances. In a more ideal situation, however, service users would have access to a drop-in center run by outreach workers, including a safe space to consume their drugs. An increasing body of evidence shows that drug consumption rooms can reduce harms and risky behavior in people who use stimulants and who smoke their drugs [31, 37]. Harms such as the spread of infectious diseases, mental health problems, and the exacerbation of social problems may be reduced through interventions offered at the DCRs, such as the distribution of safer smoking kits, education on safer drug use, access to health and social services, and the stimulation of self-control. Besides, many of the benefits of supervised injection facilities also apply to facilities for people who smoke: they provide a safe, non-rushed environment, prevent overdose fatalities, and prevent public disorders; users have access to sterile equipment; and they lead to an increase in access of social and health services [35]. In a 2017 inventory among 43 DCRs in Europe, Canada, and Australia, stimulants – including (meth)amphetamines, crack cocaine, cocaine, and cathinones – were the substances most commonly used by service users, irrespective of route of administration. Almost just as common was the use of heroin, followed by a combination of opiates and stimulants (*speedballing*). Forty-one of these DCRs offered spaces for safe injection; 31 (also) offered spaces for smoking, with 22 DCRs (also) facilitating spaces for sniffing; 34 allowed for at least 2 different means of drug administration (inject, snort, or smoke), either in separate spaces or in the same room [38]. Unfortunately, drug regulations in Indonesia do not allow for drug consumption rooms. Despite the government’s support for harm reduction measures such as NSP and OTP, the (political) likelihood of opening drug consumption rooms in the country is very low at the moment.

Finally, addressing mental health harms while reframing the connections previously established with care services was another key topic in the transition from reducing harms of opioids to reducing harms of methamphetamine use. The use of stimulants may trigger or exacerbate various mental health problems, such as anxiety, eating problems, depression, paranoia, sleep disruption, and psychotic episodes (e.g. [39, 40],). For more severe symptoms, crisis interventions by mental health professionals are recommended [6]. However, staff working with PWUS in a harm reduction setting can apply several simple techniques to provide assistance to PWUS suffering from paranoid thoughts, anxiety, or hallucinations [2, 41]. While waiting for the networking investments with mental health professionals to bear fruit, outreach workers try to meet PWUM needs by offering an attentive ear and helping users to reflect upon their perceived drug-using problems. Several service users said the support offered by the team helped them to increase self-care and self-esteem. Sharing their stories helped them to find solutions to underlying problems causing problematic use. These conversations and meetings also helped them to get more social and less isolated.

## Conclusions

### The essentials of building new harm reduction responses for methamphetamine

Recent years have seen a rise in the use of (non-injected) stimulant drugs around the world. Nevertheless, most of the harm reduction services available still focus on people who inject opioids, leaving many PWUS unassisted. Several harm reduction programs face the challenge of adapting their activities to reach non-injecting stimulant use and could greatly benefit from lessons of earlier innovative practices. Especially in Southeast Asia, the few programs which already succeeded in transitioning from assisting people injecting opioids to assisting PWUS remain unknown to the broader public; their learning processes, however, may be a tool to inspire others to build new harm reduction practices. To contribute to filling in this gap, this paper has described the learning process of a harm reduction project working with people who use methamphetamine in Jakarta, Indonesia. The project has built on previous work with people who inject opioids to reinvent itself as an outreach project that addresses the needs of PWUM. Four critical elements in this change were explored in detail. These occurred in the process of getting in touch with different types of PWUM; adapting safer smoking kits to local circumstances; and reframing partnerships with other services while addressing mental health issues as well as responding to local law enforcement practices.

An essential overarching point of the change process is that when developing an approach for promoting harm reduction to a “new” population, it is crucial to know the area, the population of PWUM, and their characteristics. The meaningful involvement of PWUM in all levels of the project – planning, running, and evaluating – was essential to making sure their perspectives were understood and included in the interventions. This occurred in the development and evaluation of outreach work, the planning and distribution of safer smoking kits, and the understanding of PWUM broader needs, including mental health care and the preferences of younger and older generations.

Additionally, pioneering a project with a population that has not been reached before requires extra effort in networking, sensitizing partners, and working toward service integration. Pioneering in a context of strict drug regulations and law enforcement also requires extra efforts and time in building trust with PWUD. This may require a compromise between maximizing the reach of the project and ensuring the quality of assistance and time needed to bond with PWUD in this initial phase.

Operating under a broad harm reduction definition was another overarching point. The aim of harm reduction is to reduce all harms associated with drug use. These may be health harms, which certainly extend beyond HIV, but also include social or economic harms such as acquisitive crime, corruption, over-incarceration, violence, stigmatization, marginalization, and harassment. This means re-centering the program on PWUM and on increasing their quality of life, rather than focusing solely on specific interventions or safer drug use supplies. Mental health care needs to receive special attention. The recognition of mental health symptoms and appropriate medical responses need to be negotiated, and PWUM perceptions of mental health symptoms need to be acknowledged, without blindly enforcing medical classifications. Furthermore, the perceived positive effects of using stimulants need to be considered when planning new harm reduction responses for these drugs.

Securing funding for harm reduction focused on people who do not inject drugs and/or who do not use opioids is fundamental in keeping programs sustainable. Increasing the investment from the national government is essential to that. Karisma has partnered with other harm reduction organizations, *Pukesmas,* and local government branches to write national guidelines on how to run harm reduction programs for PWUM. Such guidelines would make national government funding more feasible, as most of the health clinics in Jakarta only provide services in the presence of a national guideline. Once a national guideline is available, it becomes possible to budget for activities. In addition to securing national funding, encouraging international donors to step in and support harm reduction efforts without a primary focus on HIV prevention is crucial. This can help to increase the number of harm reduction projects addressing stimulant use in Indonesia and in the region. At the time of the research, Karisma’s *shabu* outreach was the only stimulant-focused harm reduction project running in Southeast Asia. Since mid-2018, a similar project has been started in Makassar, Sulawesi island, Indonesia, also supported by Mainline. The new project is integrating the lessons drawn from Jakarta. A new project focusing on reducing mental health harms of stimulants use has also started in 2019, in Vietnam.

Finally, more research is needed on the key topics of programs that need to address when transitioning from reducing harms for opiates use to reducing harms for methamphetamine. The present article builds its findings on data collected for a slightly different question, and more in-depth information could be acquired when designing research to focus specifically on this shift. Moreover, this case study addresses the shift from opiates to methamphetamine in a specific setting of strict drug policies and with a project which is a pioneer in assisting PWUM in the region. Different topics may arise as important in settings where harm reduction finds better support and projects with PWUM are further developed.

## Data Availability

The datasets used and/or analyzed during the current study are not publicly available due to individual’s anonymity reasons but are available from the corresponding author on reasonable request.

## Footnotes

1 €1 = 16.385 Indonesian rupiah

2 It is at the Compulsory Reporting Institutions that an assessment is made to determine which service the people who were reported as using drugs need.
